# Ageing under unequal circumstances: a cross-sectional analysis of the gender and socioeconomic patterning of functional limitations among the Southern European elderly

**DOI:** 10.1186/s12939-017-0673-0

**Published:** 2017-10-03

**Authors:** Manuel Serrano-Alarcón, Julian Perelman

**Affiliations:** 1 Escola Nacional de Saúde Pública, Universidade NOVA de Lisboa, Avenida Padre Cruz, 1600-560 Lisboa, Portugal; 2 Centro de Investigação em Saúde Pública, Lisboa, Portugal

**Keywords:** Functional limitations, Ageing, Inequality, Disability

## Abstract

**Background:**

In a context of population ageing, it is a priority for planning and prevention to understand the socioeconomic (SE) patterning of functional limitations and its consequences on healthcare needs. This paper aims at measuring the gender and SE inequalities in functional limitations and their age of onset among the Southern European elderly; then, we evaluate how functional status is linked to formal and informal care use.

**Methods:**

We used Portuguese, Italian and Spanish data from the Survey of Health, Ageing and Retirement in Europe (SHARE) of 2011 (*n* = 9233). We constructed a summary functional limitation score as the sum of two variables: i) Activities of Daily Living (ADL) and ii) Instrumental Activities of Daily Living (IADL). We modelled the functional limitation as a function of age, gender, education, subjective poverty, employment and marital status using multinomial logit models. We then estimated how functional limitation affected informal and formal care demand using negative binomial and logistic models.

**Results:**

Women were 2.3 percentage points (pp) more likely to experience severe functional limitation than men, and overcame a 10% probability threshold of suffering from severe limitation around 5 years earlier. Subjective poverty was associated with a 3.1 pp. higher probability of severe functional limitation. Having a university degree reduced the probability of severe functional limitation by 3.5 pp. as compared to none educational level. Discrepancies were wider for the oldest old: women aged 65-79 years old were 3.3 pp. more likely to suffer severe limitations, the excess risk increasing to 15.5 pp. among those older than 80. Similarly, educational inequalities in functional limitation were wider at older ages. Being severely limited was related with a 32.1 pp. higher probability of receiving any informal care, as compared to those moderately limited. Finally, those severely limited had on average 3.2 hospitalization days and 4.6 doctor consultations more, per year, than those without limitations.

**Conclusion:**

Functional limitations are unequally distributed, hitting women and the worse-off earlier and more severely, with consequences on care needs. Considering the burden on healthcare systems and families, public health policies should seek to reduce current inequalities in functional limitations.

**Electronic supplementary material:**

The online version of this article (10.1186/s12939-017-0673-0) contains supplementary material, which is available to authorized users.

## Background

Population ageing is a challenge for societies because of the emergence of new needs and costs. This issue is especially critical in Southern European countries: In Italy 21.7% of the population was 65 years old or over in 2015; in Portugal 20.3% and in Spain 18.5%. These are, respectively, the first, fourth and thirteenth highest rates among the EU-28 countries. At the same time, if the increasing life expectancy is not accompanied by an improvement in the functional status that offsets the ageing effect, the total number of disabled people is expected to increase. Indeed, this seems to be happening in these three countries according to the projections of The Organisation for Economic Co-operation and Development (OECD) [1].

Functional disability is a crucial issue because it imposes a high burden on health systems through long-term care (LTC), and on families when formal care is unable to address existing needs [2]. It is thus a priority to better understand the socioeconomic (SE) patterning of functional limitations in order to make accurate projections about future health and needs, and to design evidence-based prevention strategies. This paper measures the gender and SE inequalities in functional status and age of onset of disabilities amongst the Southern European elderly (Italy, Portugal, and Spain); then, we evaluate how functional status is linked to the use of formal and informal care.

There is a considerable literature showing the relationship between SE factors and functional limitations at old age. Age is an obvious predictor of functional limitations, but the age at onset of functional limitations may vary with gender and SE characteristics. First, women have been found to be more likely to start suffering earlier from functional limitations in different countries [3–6]. Second, discrepancies in functional disability between SE strata have been repeatedly found among the elderly European population, with low-educated and low-income people showing higher rates of functional disability [6, 7]. By contrast, the literature is scarce on the relationship between functional limitation and formal and informal healthcare. Bonsang E [8] found a positive relationship between the level of disability and informal care receipt, but the authors found no study on the relationship between functional limitations and healthcare demand on a comparable setting.

We first analyse functional inequalities in the specific context of Southern Europe, characterized by the above-mentioned severe ageing, coupled with strong income inequality (Spain had a Gini coefficient of 34.6 in 2015, Portugal 34, and Italy 32.4; all above the EU-28 average of 31^1^) [9] and a low coverage by formal LTC (public expenditure on LTC was 0.7% of Gross Domestic Product (GDP) for Italy and Spain in 2015, and 0.2% of GDP for Portugal, as compared to 1.1% OECD average) [10].These characteristics make these countries potentially more susceptible to experience inequalities in disability and access to LTC.

SE inequalities in functional disabilities have been studied before in Southern European countries, specifically in Greece, Italy and Spain with SHARE data from the 1st Wave 1 (2004) [11]. Our analyses uses updated data from Wave 4 (2011), in a context of economic crisis and austerity. Furthermore, we included Portugal, where these inequalities have not been studied before, to the best of our knowledge. Additionally, we analysed the age of onset of disability by gender and SE position, providing evidence on inequalities in the age of onset of functional disabilities. These findings may be relevant not only for health systems but also for retirement schemes. Finally, by estimating the link between functional limitations and (in)formal healthcare, we analysed the extent to which functional limitations are a burden on the health system and families.

## Methods

The paper used data from the Survey of Health, Ageing and Retirement in Europe (SHARE). This longitudinal database of micro data includes several health and SE indicators, among others, on individuals aged 50 and older from 20 European countries, which were collected so far through five waves (the first in 2004 and the last in 2013). We used the data from Italy, Portugal, and Spain, which were all included in the fourth wave, carried out in 2011. Response rates were 41, 43 and 62% for Italy Portugal and Spain, respectively [12]. Cross-sectional weights were used in the descriptive and multivariate analyses to control for unit non-response and to make the sample representative of the 50+ population living in residential households, following the SHARE Release Guide of Wave 4 [13]. The final sample contained 9233 individuals. The difference between this figure and the number of observations included in each model is due to individuals presenting missing values in any of the variables of the model. Note that the number of observations with such missing values accounts always for less than 8% of the (sub) sample, which makes any bias very unlikely.

### Dependent variables

We first used as dependent variable a combination of two indicators measuring functional limitations:

i) Activities of Daily Living (ADL), which indicates the number of activities for which the respondent declares to have any difficulty due to physical, mental, emotional, or memory problem. Activities included in ADL refer to dressing, bathing or showering, eating and cutting up food, walking across a room, and getting in or out of bed. There is a total of five activities. Therefore ADL varies from zero to five, according the number of activities the respondent declares to have difficulties with.
ii) Instrumental Activities of Daily Living (IADL), which relates to reported difficulties in seven relatively more complex activities: using a map, preparing a hot meal, shopping, making telephone calls, taking medications, doing work around the house and managing money. There is a total of seven instrumental activities. Therefore, IADL varies from zero to seven, according to the number of instrumental activities the respondent declares to have difficulties with.

We constructed a summary functional limitation score summing up these two variables (ADL + IADL) which goes from zero to twelve. This summary score proved to be valid for functionally disabled elderly persons [14], and has been used before to measure functional limitations in a similar setting [15, 16]. Afterwards, and in order to obtain sufficiently large groups, individuals were categorized into three levels of functional limitation: no functional limitation (ADL + IADL = 0); mild functional limitation (ADL + IADL = [1, 2]), and severe functional limitation (ADL + IADL = [3, 12]).

Second, we used informal care as dependent variable, obtained from two questions: i) existence of regular help from inside the household with personal care^2^; or ii) existence of help from any family member, friend, or neighbour from outside the household with personal help, practical household help, or help with paperwork, with a frequency of almost daily or weekly. We constructed a binary variable for informal care that was equal to one if the person received care from any of those sources, and zero otherwise.

Finally, formal healthcare utilization was measured along two dimensions: i) physician consultations (number of times the individual went to a doctor in the previous 12 months) including general practice and outpatient hospital visits; and ii) in-patient nights at hospital (number of nights spent in a hospital during the previous 12 months). These indicators have been widely used in the literature [17–19].

### Explanatory variables

Our main variables of interest were the SE factors. Wealth has been pointed out as an important SE factor indicator at older ages [16, 20]. After retirement, incomes are expected to be lower and less volatile, while wealth is an important source for current consumption at that age. Wealth can also reflect better jobs and SE status during lifetime [16, 20], and better living conditions [21]. However, the aggregate wealth variable was characterized by 42% of missing values. Furthermore, elderly people may find it difficult to precisely answer about the value of relatively complex financial assets, which were part of the wealth measurement, so that this measure may be biased. These limitations led us to consider two alternative indicators of SE condition: education level and subjective poverty. Both have been previously found to be important determinants of health inequalities among the elderly [22, 23]. Overall, 95% of the observations did not present any missing values for these two SE variables.

Education was measured by a categorical variable reflecting the highest level of education completed, following the International Standard Classification ISCED-97 from UNESCO: no education (ISCED-97 = 0), primary (ISCED-97 = 1), secondary (ISCED-97 = 2,3,4) or tertiary education (ISCED-97 = 5,6) [13]. Education can be positively linked with health through healthier lifestyles, access to better jobs, better access to health information, or higher relative position in society [24]. Furthermore, education is usually characterized by high response rates, a low respondent bias, and it is less subject to reverse causation than income [25].

An individual was labelled as poor if he declared that his household was able to make ends meet with “great” or “some difficulty”. Subjective financial well-being (i.e., subjective poverty) can be a source of stress and anxiety due to feeling of lack of control or hopelessness [23]. Furthermore, it can be an obvious constraint to access healthcare services and health promoting activities such as physical or social activities.

### Covariates

Since controlling by occupational class would be a quite restricting strategy because most part of our sample is already retired [20]; we opted instead to control by labour market status (i.e.: active, inactive or homemaker). Following previous literature [23], we also controlled for marital status with two categories: “in couple” (married or registered partnership) vs “not in couple” (Single, divorced or widowed). Finally, all analyses were controlled for age.

### Statistical analysis

We first modelled the functional limitation using a multinomial logit model, as a function of SE status, gender, age, employment status and marital status. Note that an ordered logit model seemed appropriate at first glance, but that model failed to comply with the proportional odds assumption.^3^ The ordered logit was still estimated as a robustness check and showed very similar results as compared to the multinomial logit (Additional file 1). Results of the multinomial logit were presented as marginal effects of having both moderate and severe functional limitation, respectively. Note that the reported marginal effects are adjusted for the rest of the covariates included in the same column (Tables 2 and 5). The marginal effect of an explanatory variable represents the estimated increase in the probability of suffering from functional limitation when such a variable increases by a unit, holding the rest of the covariates constant.

We further analysed whether the associations with SE factors and gender varied across age categories. First, we estimated the same model for three age subsamples: mature adults (50-65 years old), elderly (65-80 years old), and oldest old (older than 80 years). Second, we estimated average probabilities of severe limitation across age categories, by socioeconomic group (gender, education and subjective poverty), using interactions of each socioeconomic group with age categories.

Second, we estimated how the different degrees of limitation affected both informal care use and healthcare utilization. A logistic model was used to estimate how functional limitations affected the probability of receiving informal care, and marginal effects were reported, controlling by gender and SE status. Regarding healthcare use, we opted for a count data model assuming a negative binomial distribution, which was preferred to a Poisson model because of over-dispersion for both doctor visits and hospital nights [26, 27].

Analyses were performed for the full sample (pooling the three countries with country dummy variables), and for each country separately (Italy, Portugal, and Spain).

## Results

### Descriptive data

Women accounted for more than half of the population (54.4%) and the mean age was 66.5 years old (Table 1). The Portuguese sample experienced a higher prevalence of moderate or severe limitations (24.6%, compared to 21.3 and 20.7% in Spain and Italy, respectively). Portuguese people had a lower education level: 27.6% of the +50 population had secondary or tertiary education completed, as compared with 42.5% in Spain and 58.1% in Italy.

**Table 1:** Sample characteristics (weighted means and standard errors)

	ITALY		PORTUGAL		SPAIN		All	
	*n* = 3583		*n* = 2080		*n* = 3570		*n* = 9233	
VARIABLES	Mean	SD	Mean	SD	Mean	SD	Mean	SD
Female (%)	54.5	1.6	53.5	2.8	54.6	1.2	54.4	1.0
Age	66.72	0.38	65.85	0.52	66.34	0.26	66.5	0.3
Degree of functional limitation (%)								
Non limited	79.4	1.4	75.5	2.1	78.7	1.0	78.8	0.9
Moderately Limited	11.1	1.1	13.7	1.5	10.3	0.7	11.0	0.7
Severely Limited	9.6	1.1	10.9	1.6	11.0	0.7	10.2	0.6
Healthcare use								
Doctor consultations	8.46	0.31	4.96	0.41	6.99	0.22	7.59	0.19
In-patient days	1.54	0.17	1.37	0.49	1.56	0.19	1.54	0.13
Informal care (%)^a^	59.9	3.8	42.1	4.6	50.9	2.4	54.7	2.3
Education level (%)								
No education	3.0	0.6	5.1	0.8	12.6	0.7	6.8	0.4
Primary	39.0	1.6	67.3	2.3	44.8	1.2	43.7	1.0
Secondary	52.3	1.6	20.6	1.8	32.3	1.1	42.0	1.0
Tertiary	5.8	0.7	7.0	1.3	10.2	0.8	7.5	0.5
Subjective poverty (%)								
Poor	52.9	1.6	55.3	2.9	53.6	1.2	53.3	0.9
Employment status (%)								
Active	26.7	1.5	26.8	2.1	31.5	1.1	28.5	0.9
Inactive	51.3	1.6	58.5	2.8	43.1	1.2	48.9	1.0
Homemaker	22.0	1.3	14.8	2.6	25.5	1.0	22.7	0.8
Marital status (%)								
In a couple	69.5	1.6	76.7	2.1	68.6	1.2	69.8	1.0

^a^Due to questionnaire design, the informal care variable presented many missing observations between those without any functional limitation. Therefore descriptive statistics on this variable are based only on the subsample of those with one or more functional limitations

A total of 53.3% reported some or great difficulty to make ends meet, with little country variation. Regarding employment status, 31.5% of the Spanish remained active, as opposed to 26.8% in Portugal and 26.7% in Italy. Lastly, most of the subjects lived as a couple: 76.7% in Portugal, 69.5% in Italy and 68.6% in Spain.

There were wide differences across countries in the average number of doctor consultations, ranging from 4.96 in Portugal to 8.46 in Italy. In patient days however were quite similar with an average of 1.54. Lastly, among those who suffered from at least one limitation, 59.9% received informal care in Italy, 50.9% in Spain and 42.1% in Portugal (Table 1).

### Main results

On the full sample, ten additional years of age were associated with five percentage points (pp) greater likelihood of being moderately and severely functional limited (Table 2). Females were 6.1 pp. and 2.3 pp. significantly more likely to suffer from moderate and severe functional limitation, respectively, with Portugal presenting a more pronounced gender effect (7.1 pp. and 5.2 pp). Higher education levels lowered the probability of both moderately and severely limitation, globally and for each country. However, in Spain, having completed primary already significantly reduced the probability of suffering from severe functional limitation by 4.8 pp. as compared to non-education. On the contrary, in Italy and Portugal, only tertiary education significantly decreased that probability by 1.8 pp. and 4.7 pp., respectively. Having difficulties in making ends meet was significantly associated with between 5.5 pp. and 3.1 pp. higher likelihood of moderate and severe functional limitation, respectively. Again, this association was consistent across countries. Being inactive significantly increased the likelihood of functional disability, and being a homemaker only did it for Spain. Living as a couple was not associated with functional disability for a 5% significance threshold in any of the countries. Lastly, after controlling for all the variables, Italy and Spain had a significantly lower prevalence of functional limitation prevalence than Portugal.

**Table 2:** Marginal effects for functional limitation, from the multinomial model

	Moderately limited				Severely limited			
	(1)	(2)	(3)	(4)	(5)	(6)	(7)	(8)
VARIABLES	ES	IT	PT	All	ES	IT	PT	All
Age	0.004***	0.006***	0.003	0.005***	0.005***	0.003***	0.005***	0.005***
	(0.00)	(0.00)	(0.00)	(0.00)	(0.00)	(0.00)	(0.00)	(0.00)
Sex								
Base category: Male								
Female	0.047***	0.070***	0.071**	0.061***	0.032***	0.011*	0.052***	0.023***
	(0.02)	(0.02)	(0.03)	(0.01)	(0.01)	(0.01)	(0.02)	(0.01)
Education level								
Base category: *No education*								
Primary	−0.040**	−0.067	−0.034	−0.046**	−0.048***	−0.010	−0.034	−0.028***
	(0.02)	(0.04)	(0.05)	(0.02)	(0.01)	(0.01)	(0.03)	(0.01)
Secondary	−0.028	−0.108*	−0.048	−0.064***	−0.061***	−0.020	−0.028	−0.046***
	(0.02)	(0.06)	(0.05)	(0.02)	(0.01)	(0.01)	(0.02)	(0.01)
Tertiary	−0.023	−0.041	0.035	−0.021	−0.049***	−0.018***	−0.047***	−0.035***
	(0.02)	(0.04)	(0.07)	(0.02)	(0.01)	(0.01)	(0.01)	(0.01)
Subjective poverty								
Base category: *Not Poor*								
Poor	0.042***	0.056***	0.089***	0.055***	0.041***	0.015**	0.043***	0.031***
	(0.01)	(0.02)	(0.03)	(0.01)	(0.01)	(0.01)	(0.01)	(0.01)
Employment status								
Base category: *Active*								
Inactive	0.056**	0.013	0.087**	0.037**	0.026	0.032**	0.065**	0.018
	(0.03)	(0.03)	(0.04)	(0.02)	(0.02)	(0.02)	(0.03)	(0.01)
Homemaker	0.069**	0.031	0.015	0.052**	0.008	0.051	0.113	0.013
	(0.03)	(0.04)	(0.05)	(0.02)	(0.03)	(0.04)	(0.09)	(0.02)
Marital status								
Base category: *Not in a couple*								
In a couple	0.001	−0.016	0.019	−0.009	−0.014	−0.008	−0.001	−0.014*
	(0.01)	(0.02)	(0.04)	(0.01)	(0.01)	(0.01)	(0.02)	(0.01)
Country dummies								
Spain				−0.036***				−0.011
				(0.01)				(0.01)
Italy				−0.028*				−0.016*
				(0.01)				(0.01)
Observations	3222	3388	1909	8519	3222	3388	1909	8519

Standard errors in parentheses *** *p* < 0.01, ** *p* < 0.05, * *p* < 0.1. Marginal effects reported in the table are adjusted for the covariates included in the same column

The stratified analysis by age categories showed a general trend toward increasing inequalities at older ages (Table 3). First, among the oldest old (80+ years old), women had a 15.5 pp. greater probability of severe limitation compared to men. By contrast, the difference was much lower (3.3 pp) among the elderly (65-79 years old). Secondary and tertiary education reduced the likelihood of severe limitations by 1.6 pp. and 1.5 pp., respectively, among the youngest group (50-64 years old), as compared to a reduction of 21 pp. and 34.5 pp. among the oldest old. For subjective poverty, however, there was no clear age patterning in discrepancies.

**Table 3:** Marginal effects for functional limitation, from the multinomial model, by age group (full sample)

	Mature adults (50-64)		Elderly (65-79)		Oldest old (80+)	
	(1)	(2)	(3)	(4)	(5)	(6)
VARIABLES	Moderately limited	Severely limited	Moderately limited	Severely limited	Moderately limited	Severely limited
Age	0.002**	0.000	0.009***	0.009***	−0.011	0.037***
	(0.00)	(0.00)	(0.00)	(0.00)	(0.01)	(0.01)
Sex						
Base category: *Male*						
Female	0.034***	0.002	0.096***	0.033**	0.073	0.155**
	(0.01)	(0.00)	(0.02)	(0.02)	(0.05)	(0.08)
Education level						
Base category*: No education*						
Primary	−0.022	−0.005	−0.071***	−0.068***	−0.022	−0.119
	(0.02)	(0.00)	(0.03)	(0.02)	(0.10)	(0.09)
Secondary	−0.053	−0.016**	−0.060**	−0.082***	−0.069	−0.210**
	(0.04)	(0.01)	(0.02)	(0.02)	(0.09)	(0.10)
Tertiary	−0.027	−0.015***	−0.031	−0.033	0.117	−0.345***
	(0.02)	(0.00)	(0.03)	(0.03)	(0.18)	(0.08)
Subjective poverty						
Base category: *Poor*						
Poor	0.035***	0.008**	0.067***	0.075***	0.029	0.062
	(0.01)	(0.00)	(0.02)	(0.02)	(0.05)	(0.06)
Marital status						
Base category: *Not in a couple*						
In a couple	0.004	−0.005	0.005	−0.004	−0.027	−0.087
	(0.01)	(0.00)	(0.02)	(0.02)	(0.05)	(0.08)
Country dummies						
Spain	0.000	−0.017***	0.026	0.003	0.013	0.052
	(0.01)	(0.00)	(0.02)	(0.02)	(0.06)	(0.07)
Italy	0.034**	−0.001	0.047*	0.030	−0.041	0.049
	(0.02)	(0.00)	(0.03)	(0.03)	(0.08)	(0.09)
Observations	3944	3944	3577	3577	1005	1005

Standard errors in parentheses *** *p* < 0.01, ** *p* < 0.05, * *p* < 0.1. Marginal effects reported in the table are adjusted for the covariates included in the same column. Employment status was excluded of the regressions because it predicted perfectly values of the dependent variable. As a consequence convergence in the estimation of the multinomial model could not be achieved unless the employment status was dropped out

**Fig. 1:**
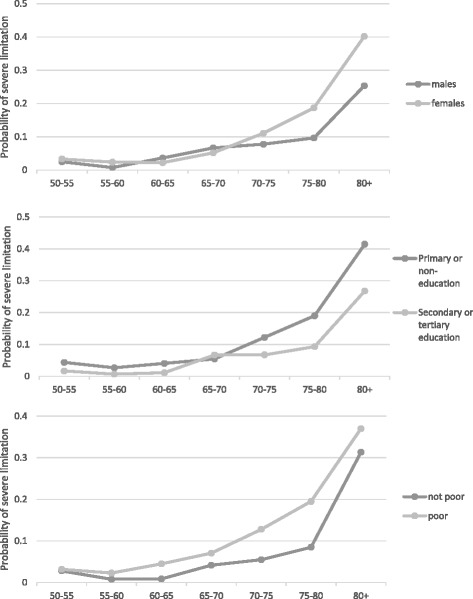
Average predicted probability of severe limitation across age categories by gender, subjective poverty status, and education level. Description of data: Predicted probabilities were calculated with the Multinomial logit model by using all observations and keeping fixed the appropriate age and socioeconomic categories. The model is similar to the one in Table 2, column (8), but including interactions with each socioeconomic group (sex, education or subjective poverty) and 5-years age dummies

The risk of suffering from severe functional limitation rose dramatically for women, and started to diverge from that of the men, after 65-70 years old (Fig. 1). For men, that risk reaches 10% at 75–80 years old, while by that age the risk for women was almost 20%. There were no marked differences in the average probabilities across education categories for the youngest age categories; however, after 65 years old the average probability of severe functional limitation for the low educated increased dramatically to above 40% at 80+ years old, whereas it was 26% among the high education category. Disparities by poverty status follow a slightly different pattern: they start earlier (at 55-60 years old), reaching the highest point at around 75-80 years old, and then they tend to diminish at 80+ years old.

Being severely limited increased the likelihood of receiving informal care by 32.1 pp., compared to those with moderate limitations, with consistent results across countries (35.8 pp. in Italy, 33 pp. in Portugal and 25.5 pp. in Spain) (Table 4). Italy showed a significantly higher provision of informal care.

**Table 4:** Marginal effects from the logistic model of informal care, among those limited

	(1)	(2)	(3)	(4)
VARIABLES	ES	IT	PT	All
Level of limitation				
Base category: *Moderately limited*				
Severely Limited	0.255***	0.358***	0.330***	0.321***
	(0.04)	(0.04)	(0.04)	(0.03)
Age	0.009***	0.005*	0.010***	0.008***
	(0.00)	(0.00)	(0.00)	(0.00)
Sex				
Base category: *Male*				
Female	0.073	−0.092	0.067	−0.024
	(0.06)	(0.07)	(0.08)	(0.05)
Education level				
Base category: *No education*				
Primary	0.008	0.205**	0.165**	0.088
	(0.05)	(0.09)	(0.08)	(0.05)
Secondary	−0.124*	0.263***	0.243**	0.084
	(0.07)	(0.10)	(0.11)	(0.06)
Tertiary	−0.073	−0.056	0.173	−0.056
	(0.12)	(0.16)	(0.17)	(0.12)
Subjective poverty				
Base category: *Not poor*				
poor	−0.017	0.032	0.018	0.003
	(0.05)	(0.06)	(0.07)	(0.04)
Employment status				
Base category: *Active*				
Inactive	0.165	−0.026	−0.006	0.094
	(0.11)	(0.13)	(0.13)	(0.08)
Homemaker	0.046	0.089	0.059	0.116
	(0.12)	(0.15)	(0.14)	(0.09)
Marital status				
Base category: *Not in a couple*				
In a couple	0.066	−0.043	−0.029	0.007
	(0.05)	(0.06)	(0.07)	(0.04)
Country dummies				
Spain				0.054
				(0.04)
Italy				0.115**
				(0.05)
Observations	727	603	504	1834

Standard errors in parentheses *** *p* < 0.01, ** *p* < 0.05, * *p* < 0.11. Average marginal effects from the logistic model of informal care. The informal care among those with no functional limitation only concerned very few people, while there were many missing observations, so individuals with no functional limitation were dropped from this analysis

**Fig. 2:**
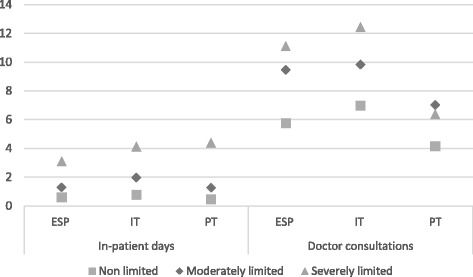
Predicted in-patient days and doctor consultations from the models of healthcare use (Table 5). Predicted values were calculated at each level of functional limitation, holding the rest of the explanatory variables at their means, and independently for each country

Lastly, both degrees of functional limitation (moderate and severe) were significantly positively correlated with the number of consultations and in-patient days, after controlling for SE characteristics (Table 5). The predicted number of in-patient days was below one for non-limited people in the three countries, as compared to 3.1 days in Spain and around 4 days in Italy and Portugal, for those with severe limitations. The predicted number of consultations showed a significant variation across countries and limitation categories. In Portugal, they varied between 4.14 for the non-limited and 7.2 for the severely limited; in Spain from 5.7 to 11.1, and in Italy from 7 to 12.4 (Fig. 2). Finally, the main results presented in this section were robust to sensitivity analysis with a lower and a higher cut-off to distinguish between moderate and severe limitation (Additional files 2, 3, 4, 5, 6, 7 and 8).

**Table 5:** Coefficients of the negative binomial models of healthcare use

	In-patient days				Doctor consultations			
	(1)	(2)	(3)	(4)	(5)	(6)	(7)	(8)
VARIABLES	ES	IT	PT	All	ES	IT	PT	All
Level of limitation								
Base category: *Non-limited*								
Moderately Limited	0.750**	0.928***	0.979***	0.799***	0.500***	0.344***	0.529***	0.419***
	(0.33)	(0.32)	(0.36)	(0.21)	(0.08)	(0.11)	(0.14)	(0.06)
Severely Limited	1.625***	1.663***	2.220***	1.733***	0.660***	0.580***	0.431**	0.604***
	(0.28)	(0.27)	(0.48)	(0.20)	(0.09)	(0.12)	(0.17)	(0.08)
Sex								
Base category: *Male*								
Female	−0.550**	−0.066	0.275	−0.234	0.186**	0.101	0.364**	0.154***
	(0.28)	(0.28)	(0.30)	(0.20)	(0.08)	(0.07)	(0.15)	(0.05)
Age	−0.010	0.032**	0.023	0.010	−0.006	0.005	0.003	0.001
	(0.01)	(0.01)	(0.02)	(0.01)	(0.00)	(0.00)	(0.01)	(0.00)
Education level								
Base category: *No education*								
Primary	−0.086	0.467	0.469	−0.039	0.058	0.039	0.169	0.095
	(0.29)	(0.47)	(0.54)	(0.24)	(0.08)	(0.12)	(0.18)	(0.07)
Secondary	−0.653**	0.290	0.981	−0.296	−0.093	−0.149	0.191	−0.070
	(0.32)	(0.50)	(0.64)	(0.26)	(0.09)	(0.14)	(0.20)	(0.08)
Tertiary	−0.758	0.423	0.907	−0.197	−0.043	−0.229	0.482	−0.025
	(0.55)	(0.70)	(0.66)	(0.43)	(0.17)	(0.20)	(0.30)	(0.12)
Subjective poverty								
Base category: *Not poor*								
poor	0.289	0.078	0.442	0.273	0.104	0.169**	0.011	0.133***
	(0.26)	(0.21)	(0.31)	(0.17)	(0.07)	(0.07)	(0.17)	(0.05)
Employment status								
Base category: *Active*								
Inactive	1.711***	0.332	0.607	0.892***	0.537***	0.552***	0.218	0.516***
	(0.37)	(0.35)	(0.39)	(0.25)	(0.11)	(0.10)	(0.18)	(0.07)
Homemaker	1.790***	0.031	0.404	0.708**	0.333***	0.365***	0.354	0.339***
	(0.46)	(0.44)	(0.56)	(0.30)	(0.11)	(0.12)	(0.30)	(0.08)
Marital status								
Base category: *Not in a couple*								
In a couple	−0.387	0.223	−0.121	−0.022	0.142**	0.041	0.039	0.078
	(0.27)	(0.23)	(0.36)	(0.18)	(0.07)	(0.08)	(0.12)	(0.05)
Country dummies								
Spain FE				0.231				0.400***
				(0.22)				(0.09)
Italy FE				0.425*				0.559***
				(0.22)				(0.09)
Observations	3212	3384	1903	8499	3203	3372	1881	8456

Standard errors in parentheses *** *p* < 0.01, ** *p* < 0.05, * *p* < 0.1

## Discussion

### Key results

First, results showed that more than 20% of the Southern European people older than 50 suffered from functional limitations. Second, gender and SE status were important predictors of severe functional limitation, with a 2.3 pp. higher risk among women, and a 3.5 pp. lower risk among those with tertiary education. Third, gender and SE disparities in functional limitations increased with age. Around 65-70 years old, the risk of suffering from severe functional limitation dramatically increased for women and low-educated individuals, this was also the case at 55-60 years old for those declaring to have difficulties to make ends meet. Fourth, the probability of receiving informal and formal care was greater for the severely limited than for the moderately limited. All these results were quite consistent across the three Southern Europe countries included in our sample.

### Interpretations

In line with the literature, women were more likely to suffer from functional limitations [3–6]. It has been suggested that this finding derives from many factors: unfavourable biological characteristics, greater propensity to admit illness, or different social roles and relationships that make women more vulnerable to poor health [28, 29]. According to our results, this is amongst the most important factor to explain functional disability, jointly with education, subjective poverty and age.

The negative relationship between SE strata and functional limitations also confirms earlier reported evidence [6, 7, 23]. Education seems to have a very important effect on the chances of suffering from functional limitations. Channels may be healthier behaviours, better jobs during the lifetime, access to better health information, or a higher relative position in society. Subjective poverty is more likely to reflect current financial constraints that may affect mental health or access to health-related services.

Earlier studies have found smaller educational and income differences in health at older ages [22, 30]. However, our findings are in the opposite direction, with gender and education disparities in functional disabilities being greater amongst the oldest old. The authors explain the increasing inequality by a postponement in morbidity by those of high SE status, and by selective mortality, which was however not supported by the data [22]. In our view, two explanations can be put forward to justify why our results differ from the literature. First, we focused on functional limitations, while the literature examines specific diseases. Even if the prevalence of diseases becomes more equally distributed among the oldest old, the “experience” of disease may still vary with SE factors. The better-off may experience a lower or slower deterioration of health, for example due to better access to care and other psychosocial resources, with consequences on dependency. A second explanation is that Southern Europe has been experiencing dramatic and fast changes over the twentieth century, which may have generated strong differences across cohorts. People older than 80 lived their childhood in the years 1930-1940, when Italy, Spain and Portugal were poor rural countries, marked by huge inequalities. This is a different context from that of the 1960s, experienced by the youngest people in our sample, marked by a catching-up of the economy and the implementation of social welfare.

The number of functional limitations is significantly and positively related with the probability of receiving informal care, highlighting that our measure of functional limitations is a good proxy for dependence. In a strict sense, dependence implies the need of help from someone else in doing daily activities [31]. By contrast, reported difficulties denote that the person carries out one activity with some problem, but does not necessarily indicate the need for help from a third person [32]. However, in practical terms, dependence has usually been measured using reported difficulties in ADL and IADL, when data about the need for help was not available [33]. In that sense, we can consider the occurrence of functional limitations as a first stage in the onset of dependence and a valid proxy for dependency, as Grammenos S [32] argues.

Note finally that the Portuguese 50+ population showed a relatively worse functional status as compared to the Spanish and the Italian, even after controlling for all the SE characteristics. At the same time, the level of both informal care and healthcare use is the highest in Italy and the lowest in Portugal, even after controlling for functional status and SE factors. Considering the lowest level of public long-term care expenditure in Portugal (0.2 GDP vs 0.7% GDP in Spain and Italy) [10], these results suggest the existence of potential unmet needs among the functionally disabled Portuguese, as compared to their Southern European counterparts.

### Implications

Our findings have several possible societal implications. First, women and those with a lower SE status suffer the onset of functional disability at younger ages than other categories. These disparities in functional limitations may imply a high burden on retirement schemes. Individuals at higher risk of functional disability may become unable to work at younger ages and will have to rely on other sources of income (e.g., public pension). This finding highlights the need for prevention policies that more specifically target the most vulnerable people, those more at risk of becoming early disabled.

Second, the higher needs of informal care amongst those with functional limitations impose a greater burden on their relatives. In that sense, since a substitution relationship between formal and informal care is expected among the most severely dependent people [19], the limited expansion of LTC in Southern Europe may be an important factor explaining the high degree of correlation between the number of functional limitations and the probability of receiving informal care.

The fact that women are more affected by functional limitation may put additional pressure on other family members rather than the partner, who can be thought as the “natural” caregiver. Women live longer and are likely to outlive spouses. Therefore, those women with functional limitations will have to rely on other family members in the absence of the partner. In that sense, the promotion of LTC seems key to address the needs not only of the dependents, but also of their relatives. However, it is worth noting that the high correlation between functional limitations and informal care may have cultural explanations as well, since Mediterranean societies have closer family relations than northern European countries [34]. Nevertheless, such close relations might be expected to change due to smaller size households, lower birth rates and higher geographical mobility of the descendants.

The greater disability amongst the worse-off is also problematic because access to formal LTC is limited in Southern Europe, especially in Portugal, except for those who can afford private facilities. It is thus expectable that the worse-off may also rely more on informal care, which families may have difficulties in providing. The inequalities in disability may thus also provoke a rise in inequalities in the access to high-quality LTC.

Finally, a higher prevalence of functional limitations will increase the pressure on public health systems due to the positive association between functional disability and healthcare demand. This may be reinforced by the fact that those with the lowest SE status (and highest functional limitation) are less likely to have private health insurance, and rely only on public health services [35].

### Limitations

This study presents some limitations that should be taken into account. First of all, we did not use longitudinal data, so our findings are not immune to reverse causation and unobserved heterogeneity. The reverse causation may exist in regard to subjective poverty, which may be caused by functional limitations. Note, however, that poverty at old ages is very likely to have been determined in the past, before the onset of disability. In regard to unobserved heterogeneity, it is likely that low SE status and functional limitations are caused by common past factors, such as childhood health and family background. SHARE data, from Wave 4, did not include questions about life history, so that these issues could not be addressed. Note that our goal was to depict the patterning of functional limitations for future planning however, and not a measurement of detailed causal relationships.

Furthermore, due to the lack of panel data from the three countries, we were not able to determine whether the wider disparities found at older ages were actually due to ageing, or on the contrary, a consequence of cohort effects. We expect that this analysis will be improved by new waves of the SHARE database including all of them.

Finally, the analysis also lacks data about formal LTC, which was not asked in the Wave 4 of SHARE. Nevertheless, we know from the OECD data that the LTC system is underdeveloped in these countries as compared to other European countries [10]. So, even if the survey included that data, LTC would not be expected to be very widespread across the Southern European population.

## Conclusions

In a context of ageing and high income inequalities, 25% of the Portuguese and around 21% of the Italian and Spanish people older than 50 suffered from functional limitation. These results clearly point out that dependency amongst the elderly should be a public health priority in these countries. Also, functional disabilities are unequally distributed, hitting earlier and more severely women and the worse-off, with potentially dramatic consequences on families, social security schemes, and health systems. Public health policies should thus aim to reduce disabilities and their early onset, and also the inequalities in disability, by developing prevention strategies oriented toward the most vulnerable.

## Additional files

Additional file 1: Coefficients of ordered logit model for functional limitations. Robustness check (I) of Table 2. Standard errors in parentheses *** *p* < 0.01, ** *p* < 0.05, * *p* < 0.10. The dependent variable represents three levels of functional limitation: no functional limitation (ADL + IADL = 0); mild functional limitation (ADL + IADL = [1, 2]), and severe functional limitation (ADL + IADL = [3, 12]). (DOCX 13 kb)

Additional file 2: Marginal effects for functional limitation from the multinomial model. Robustness check (II) of Table 2. Standard errors in parentheses *** *p* < 0.01, ** *p* < 0.05, * *p* < 0.1. Estimation of the same model as in Table 2, but setting a new cut-off for the dependent variable of functional limitation: moderately functionally limited if ADL + IADL equals one and severely functionally limited if ADL + IADL is equal or greater than two. (DOCX 16 kb)

Additional file 3: Marginal effects for functional limitation from the multinomial model. Robustness check (III) of Table 2. Standard errors in parentheses *** *p* < 0.01, ** *p* < 0.05, * *p* < 0.1. Estimation of the same model as in Table 2, but setting a new cut-off for the dependent variable of functional limitation: moderately functionally limited if ADL + IADL is between one and three and severely functionally limited if ADL + IADL is equal or greater than four. (DOCX 16 kb)

Additional file 4: Marginal effects for functional limitation from the multinomial model, by age group. Robustness check (I) of Table 3. Standard errors in parentheses *** *p* < 0.01, ** *p* < 0.05, * *p* < 0.1. Estimation of the same model as in Table 3, but setting a new cut-off for the dependent variable of functional limitation: moderate functionally limited if ADL + IADL equals one and severe functionally limited if ADL + IADL is equal or greater than two. (DOCX 14 kb)

Additional file 5: Marginal effects for functional limitation from the multinomial model, by age group. Robustness check (II) of Table 3. Standard errors in parentheses *** *p* < 0.01, ** *p* < 0.05, * *p* < 0.1. Estimation of the same model as in Table 3, but setting a new cut-off for the dependent variable of functional limitation: moderate functionally limited if ADL + IADL is between one and three and severe functionally limited if ADL + IADL is equal or greater than four. (DOCX 14 kb)

Additional file 6: Marginal effects from the logistic model of informal care, among those limited. Robustness check of Table 4. Standard errors in parentheses *** *p* < 0.01, ** *p* < 0.05, * *p* < 0.1. The new cut-offs for the level of limitation were: For columns (1) to (4) moderate functionally limited if ADL + IADL equals one and severe functionally limited if ADL + IADL is equal or greater than two; for columns (5) to (8) moderate functionally limited if ADL + IADL is between one and three and severe functionally limited if ADL + IADL is equal or greater than four. (DOCX 16 kb)

Additional file 7: Coefficients of the negative binomial models of healthcare use. Robustness check (I) of Table 5. Standard errors in parentheses *** *p* < 0.01, ** *p* < 0.05, * *p* < 0.1. The new cut-offs for the level of limitation were: moderate functionally limited if ADL + IADL equals one and severe functionally limited if ADL + IADL is equal or greater than two. (DOCX 17 kb)

Additional file 8: Coefficients of the negative binomial models of healthcare use. Robustness check (II) of Table 5. Standard errors in parentheses *** *p* < 0.01, ** *p* < 0.05, * *p* < 0.1. The new cut-offs for the level of limitation were: moderate functionally limited if ADL + IADL is between one and three and severe functionally limited if ADL + IADL is equal or greater than four. (DOCX 18 kb)

## Abbreviations

ADL: Activities of daily living
ES: Spain
GDP: Gross domestic product
IADL: Instrumental activities of daily living
IT: Italy
LTC: Long-term care
OECD: Organisation for economic and development
pp.: Percentage points
PT: Portugal
SE: Socioeconomic
SHARE: Survey of Health, Ageing and Retirement in Europe
SNS: Portuguese Nacional Health Service

## Footnotes

1 Note that Gini goes from 0 (perfect equality) to 100 (maximal inequality).

2 By regularly we mean “daily or almost daily during at least 3 months in the last 12 months”.

3 For further explanation about the proportional odds assumption. https://stats.idre.ucla.edu/stata/dae/ordered-logistic-regression/ Last visited 26/07/2017
