# Supplementary material for: Ageing under unequal circumstances: a cross-sectional analysis of the gender and socioeconomic patterning of functional limitations among the Southern European elderly
Source: Int J Equity Health. 2017 Oct 3;16:175. doi: 10.1186/s12939-017-0673-0 (PMC5627490; doi:10.1186/s12939-017-0673-0)
Supplement: Supplementary file 1 — Coefficients of ordered logit model for functional limitations. Robustness check (I) of Table 2. Standard errors in parentheses *** p < 0.01, ** p < 0.05, * p < 0.10. The dependent variable represents three levels of functional limitation: no functional limitation (ADL + IADL = 0); mild functional limitation (ADL + IADL = [1, 2]), and severe functional limitation (ADL + IADL = [3, 12]). (DOCX 13 kb) [file 12939_2017_673_MOESM1_ESM.docx]

|  | (1) | (2) | (3) | (4) |
| --- | --- | --- | --- | --- |
| VARIABLES | ES | IT | PT | All |
|  |  |  |  |  |
| Age | 0.082*** | 0.120*** | 0.069*** | 0.098*** |
|  | (0.01) | (0.01) | (0.02) | (0.01) |
| Sex | |  |  |  |
| Base category: *Male* | |  |  |  |
| female | 0.646*** | 0.768*** | 0.924*** | 0.722*** |
|  | (0.17) | (0.24) | (0.24) | (0.14) |
| Education level | |  |  |  |
| Base category: *No education* | |  |  |  |
| Primary | -0.765*** | -0.452 | -0.578 | -0.626*** |
|  | (0.15) | (0.47) | (0.40) | (0.16) |
| Secondary | -0.864*** | -0.930* | -0.729 | -0.991*** |
|  | (0.19) | (0.51) | (0.50) | (0.19) |
| Tertiary | -0.893*** | -1.081* | -0.479 | -0.902*** |
|  | (0.31) | (0.57) | (0.48) | (0.24) |
| Subjective poverty | |  |  |  |
| Base category: *Not poor* | |  |  |  |
| Poor | 0.670*** | 0.675*** | 0.948*** | 0.720*** |
|  | (0.13) | (0.18) | (0.24) | (0.11) |
| Employment status | |  |  |  |
| Base category: *Active* | |  |  |  |
| Inactive | 0.595** | 0.076 | 0.785** | 0.361* |
|  | (0.27) | (0.34) | (0.31) | (0.19) |
| Homemaker | 0.421 | 0.165 | 0.502 | 0.304 |
|  | (0.27) | (0.37) | (0.43) | (0.20) |
| Marital status | |  |  |  |
| Based category: *Not in a couple* | |  |  |  |
| In a couple | -0.101 | -0.259 | 0.015 | -0.191 |
|  | (0.15) | (0.20) | (0.29) | (0.12) |
|  |  |  |  |  |
| Country dummies |  |  |  |  |
| Spain |  |  |  | -0.379*** |
|  |  |  |  | (0.14) |
| Italy |  |  |  | -0.403*** |
|  |  |  |  | (0.15) |
| Observations | 3222 | 3388 | 1909 | 8519 |
|  |  |  |  |  |
