# Supplementary material for: Ageing under unequal circumstances: a cross-sectional analysis of the gender and socioeconomic patterning of functional limitations among the Southern European elderly
Source: Int J Equity Health. 2017 Oct 3;16:175. doi: 10.1186/s12939-017-0673-0 (PMC5627490; doi:10.1186/s12939-017-0673-0)
Supplement: Supplementary file 2 — Marginal effects for functional limitation from the multinomial model. Robustness check (II) of Table 2. Standard errors in parentheses *** p < 0.01, ** p < 0.05, * p < 0.1. Estimation of the same model as in Table 2, but setting a new cut-off for the dependent variable of functional limitation: moderately functionally limited if ADL + IADL equals one and severely functionally limited if ADL + IADL is equal or greater than two. (DOCX 16 kb) [file 12939_2017_673_MOESM2_ESM.docx]

|  | | | Moderately limited | | | |  | Severely limited | | | |  |  |
| --- | --- | --- | --- | --- | --- | --- | --- | --- | --- | --- | --- | --- | --- |
|  | | | (1) | (2) | (3) | (4) |  | (5) | (6) | (7) | (8) |  |  |
| VARIABLES | | | ES | IT | PT | All |  | ES | IT | PT | All |  |  |
|  | | |  |  |  |  |  |  |  |  |  |  |  |
| Age | | | 0.003*** | 0.003*** | 0.000 | 0.003*** |  | 0.007*** | 0.006*** | 0.007*** | 0.007*** |  |  |
|  | | | (0.00) | (0.00) | (0.00) | (0.00) |  | (0.00) | (0.00) | (0.00) | (0.00) |  |  |
| Sex | | | |  |  |  |  |  |  |  |  |  |  |
| Base category: *Male* | | | |  |  |  |  |  |  |  |  |  |  |
| female | | | 0.033** | 0.039** | 0.037 | 0.037*** |  | 0.046*** | 0.028*** | 0.090*** | 0.042*** |  |  |
|  | | | (0.01) | (0.02) | (0.02) | (0.01) |  | (0.02) | (0.01) | (0.02) | (0.01) |  |  |
| Education level | | | |  |  |  |  |  |  |  |  |  |  |
| Base category: *No education* | | | | |  |  |  |  |  |  |  |  |  |
| Primary | | | -0.026** | -0.049 | -0.023 | -0.031** |  | -0.063*** | -0.025 | -0.056 | -0.043*** |  |  |
|  | | | (0.01) | (0.03) | (0.04) | (0.01) |  | (0.01) | (0.02) | (0.04) | (0.01) |  |  |
| Secondary | | | -0.015 | -0.074 | -0.035 | -0.040** |  | -0.074*** | -0.046* | -0.049 | -0.068*** |  |  |
|  | | | (0.01) | (0.05) | (0.04) | (0.02) |  | (0.02) | (0.02) | (0.03) | (0.01) |  |  |
| Tertiary | | | -0.004 | -0.012 | -0.024 | -0.002 |  | -0.067*** | -0.039*** | -0.010 | -0.053*** |  |  |
|  | | | (0.02) | (0.05) | (0.04) | (0.03) |  | (0.02) | (0.01) | (0.05) | (0.01) |  |  |
|  | | |  |  |  |  |  |  |  |  |  |  |  |
| Base category: *Not poor* | | | | |  |  |  |  |  |  |  |  |  |
| Poor | | | 0.031*** | 0.040*** | 0.062** | 0.040*** |  | 0.053*** | 0.029*** | 0.072*** | 0.045*** |  |  |
|  | | | (0.01) | (0.01) | (0.03) | (0.01) |  | (0.01) | (0.01) | (0.02) | (0.01) |  |  |
| Employment status | | | | |  |  |  |  |  |  |  |  |  |
| Base category: *Active* | | | | |  |  |  |  |  |  |  |  |  |
| Inactive | | | 0.023 | 0.029 | 0.068* | 0.030* |  | 0.062** | 0.025 | 0.077** | 0.032** |  |  |
|  | | | (0.02) | (0.02) | (0.04) | (0.02) |  | (0.03) | (0.02) | (0.03) | (0.02) |  |  |
| Homemaker | | | 0.030 | 0.047 | 0.006 | 0.041** |  | 0.048 | 0.035 | 0.099 | 0.030 |  |  |
|  | | | (0.03) | (0.03) | (0.04) | (0.02) |  | (0.03) | (0.03) | (0.07) | (0.02) |  |  |
| Cohabitation | | | | |  |  |  |  |  |  |  |  |  |
| Based category: *Not in a couple* | | | | |  |  |  |  |  |  |  |  |  |
| In a couple | | | 0.010 | 0.016 | 0.004 | 0.012 |  | -0.022 | -0.027** | 0.009 | -0.029*** |  |  |
|  | | | (0.01) | (0.01) | (0.04) | (0.01) |  | (0.02) | (0.01) | (0.03) | (0.01) |  |  |
|  | | |  |  |  |  |  |  |  |  |  |  |  |
| Country dummies | | | |  |  |  |  |  |  |  |  |  |  |
| Spain | | |  |  |  | -0.030*** |  |  |  |  | -0.016 |  |  |
|  | | |  |  |  | (0.01) |  |  |  |  | (0.01) |  |  |
| Italy | | |  |  |  | -0.024** |  |  |  |  | -0.019* |  |  |
|  | | |  |  |  | (0.01) |  |  |  |  | (0.01) |  |  |
| Observations | | | 3233 | 3390 | 1912 | 8525 |  | 3223 | 3390 | 1912 | 8525 |  |  |
|  | | |  |  |  |  |  |  |  |  |  |  |  |
|  |  |  |  |  |  |  |  |  |  |  |  |  |  |
