# Supplementary material for: Ageing under unequal circumstances: a cross-sectional analysis of the gender and socioeconomic patterning of functional limitations among the Southern European elderly
Source: Int J Equity Health. 2017 Oct 3;16:175. doi: 10.1186/s12939-017-0673-0 (PMC5627490; doi:10.1186/s12939-017-0673-0)
Supplement: Supplementary file 3 — Marginal effects for functional limitation from the multinomial model. Robustness check (III) of Table 2. Standard errors in parentheses *** p < 0.01, ** p < 0.05, * p < 0.1. Estimation of the same model as in Table 2, but setting a new cut-off for the dependent variable of functional limitation: moderately functionally limited if ADL + IADL is between one and three and severely functionally limited if ADL + IADL is equal or greater than four. (DOCX 16 kb) [file 12939_2017_673_MOESM3_ESM.docx]

|  | Moderately limited | | | |  | Severely limited | | | |  |
| --- | --- | --- | --- | --- | --- | --- | --- | --- | --- | --- |
|  | (1) | (2) | (3) | (4) |  | (5) | (6) | (7) | (8) |  |
| VARIABLES | ES | IT | PT | All |  | ES | IT | PT | All |  |
|  |  |  |  |  |  |  |  |  |  |  |
| Age | 0.005*** | 0.008*** | 0.003 | 0.006*** |  | 0.005*** | 0.002*** | 0.003*** | 0.004*** |  |
|  | (0.00) | (0.00) | (0.00) | (0.00) |  | (0.00) | (0.00) | (0.00) | (0.00) |  |
| Sex | |  |  |  |  |  |  |  |  |  |
| Base category: *Male* | |  |  |  |  |  |  |  |  |  |
| female | 0.055*** | 0.087*** | 0.076** | 0.074*** |  | 0.024** | 0.001 | 0.040*** | 0.012* |  |
|  | (0.02) | (0.02) | (0.03) | (0.01) |  | (0.01) | (0.00) | (0.02) | (0.01) |  |
| Education level | |  |  |  |  |  |  |  |  |  |
| Base category: *No education* | |  |  |  |  |  |  |  |  |  |
| Primary | -0.058*** | -0.063 | -0.109* | -0.055*** |  | -0.034*** | -0.008 | -0.000 | -0.022*** |  |
|  | (0.02) | (0.05) | (0.06) | (0.02) |  | (0.01) | (0.01) | (0.01) | (0.01) |  |
| Secondary | -0.045*** | -0.110* | -0.080* | -0.080*** |  | -0.048*** | -0.012 | -0.016 | -0.034*** |  |
|  | (0.02) | (0.06) | (0.05) | (0.02) |  | (0.01) | (0.01) | (0.01) | (0.01) |  |
| Tertiary | -0.034 | -0.045 | -0.030 | -0.032 |  | -0.042*** | -0.011*** | -0.021* | -0.030*** |  |
|  | (0.02) | (0.04) | (0.06) | (0.02) |  | (0.01) | (0.00) | (0.01) | (0.01) |  |
| Subjective poverty | |  |  |  |  |  |  |  |  |  |
| Base category: *Not poor* | |  |  |  |  |  |  |  |  |  |
| Poor | 0.049*** | 0.062*** | 0.102*** | 0.062*** |  | 0.034*** | 0.008** | 0.026** | 0.025*** |  |
|  | (0.01) | (0.02) | (0.03) | (0.01) |  | (0.01) | (0.00) | (0.01) | (0.01) |  |
| Employment status | |  |  |  |  |  |  |  |  |  |
| Base category: *Active* | |  |  |  |  |  |  |  |  |  |
| Inactive | 0.081*** | 0.007 | 0.118*** | 0.048** |  | 0.001 | 0.053** | 0.028 | 0.006 |  |
|  | (0.03) | (0.03) | (0.04) | (0.02) |  | (0.02) | (0.02) | (0.02) | (0.01) |  |
| Homemaker | 0.101*** | 0.007 | 0.074 | 0.060** |  | -0.017 | 0.157 | 0.037 | 0.003 |  |
|  | (0.04) | (0.03) | (0.07) | (0.03) |  | (0.02) | (0.10) | (0.05) | (0.01) |  |
| Marital status | |  |  |  |  |  |  |  |  |  |
| Base category: *Not in a couple* | | |  |  |  |  |  |  |  |  |
| In a couple | -0.002 | -0.023 | 0.023 | -0.014 |  | -0.012 | -0.003 | 0.000 | -0.010 |  |
|  | (0.02) | (0.02) | (0.04) | (0.01) |  | (0.01) | (0.00) | (0.01) | (0.01) |  |
|  |  |  |  |  |  |  |  |  |  |  |
| Country dummies | |  |  |  |  |  |  |  |  |  |
| Spain |  |  |  | -0.039*** |  |  |  |  | -0.008 |  |
|  |  |  |  | (0.01) |  |  |  |  | (0.01) |  |
| Italy |  |  |  | -0.028* |  |  |  |  | -0.014* |  |
|  |  |  |  | (0.02) |  |  |  |  | (0.01) |  |
|  |  |  |  |  |  |  |  |  |  |  |
| Observations | 3223 | 3390 | 1912 | 8525 |  | 3223 | 3390 | 1912 | 8525 |  |
|  |  |  |  |  |  |  |  |  |  |  |
