# Supplementary material for: Ageing under unequal circumstances: a cross-sectional analysis of the gender and socioeconomic patterning of functional limitations among the Southern European elderly
Source: Int J Equity Health. 2017 Oct 3;16:175. doi: 10.1186/s12939-017-0673-0 (PMC5627490; doi:10.1186/s12939-017-0673-0)
Supplement: Supplementary file 4 — Marginal effects for functional limitation from the multinomial model, by age group. Robustness check (I) of Table 3. Standard errors in parentheses *** p < 0.01, ** p < 0.05, * p < 0.1. Estimation of the same model as in Table 3, but setting a new cut-off for the dependent variable of functional limitation: moderate functionally limited if ADL + IADL equals one and severe functionally limited if ADL + IADL is equal or greater than two. (DOCX 14 kb) [file 12939_2017_673_MOESM4_ESM.docx]

|  | Mature adults (50-64) | | Elderly (65-79) | | Oldest old (80+) | |
| --- | --- | --- | --- | --- | --- | --- |
|  | (1) | (2) | (3) | (4) | (5) | (6) |
| VARIABLES | Moderately  limited | Severely  limited | Moderately  limited | Severely  limited | Moderately  limited | Severely  limited |
|  |  |  |  |  |  |  |
| Age | 0.002** | 0.000 | 0.004*** | 0.014*** | -0.005 | 0.034*** |
|  | (0.00) | (0.00) | (0.00) | (0.00) | (0.00) | (0.01) |
| Sex |  |  |  |  |  |  |
| Base category: *Male* |  |  |  |  |  |  |
| Female | 0.025*** | 0.009* | 0.059*** | 0.061*** | 0.011 | 0.226*** |
|  | (0.01) | (0.00) | (0.02) | (0.02) | (0.03) | (0.08) |
| Education level |  |  |  |  |  |  |
| Base category*: No education* | |  |  |  |  |  |
| Primary | -0.019 | -0.009 | -0.070*** | -0.078*** | 0.052 | -0.197*** |
|  | (0.02) | (0.01) | (0.02) | (0.03) | (0.04) | (0.08) |
| Secondary | -0.039 | -0.033*** | -0.050*** | -0.094*** | -0.006 | -0.268*** |
|  | (0.03) | (0.01) | (0.02) | (0.02) | (0.06) | (0.10) |
| Tertiary | -0.024 | -0.021*** | -0.039* | -0.033 | 0.350* | -0.504*** |
|  | (0.02) | (0.00) | (0.02) | (0.04) | (0.20) | (0.06) |
| Subjective poverty |  |  |  |  |  |  |
| Base category: *Not poor* | |  |  |  |  |  |
| Poor | 0.030*** | 0.014*** | 0.040*** | 0.100*** | 0.008 | 0.090 |
|  | (0.01) | (0.01) | (0.02) | (0.02) | (0.04) | (0.06) |
| Marital status |  |  |  |  |  |  |
| Based category: *Not in a couple* | |  |  |  |  |  |
| In a couple | 0.001 | -0.004 | 0.030* | -0.024 | 0.045 | -0.165** |
|  | (0.01) | (0.01) | (0.02) | (0.02) | (0.04) | (0.07) |
|  |  |  |  |  |  |  |
| Country dummies |  |  |  |  |  |  |
| Spain | -0.027*** | 0.001 | -0.033** | -0.037 | 0.017 | -0.025 |
|  | (0.01) | (0.01) | (0.02) | (0.02) | (0.07) | (0.09) |
| Italy | -0.022* | -0.024*** | -0.005 | -0.031 | -0.029 | 0.096 |
|  | (0.01) | (0.01) | (0.02) | (0.03) | (0.07) | (0.10) |
|  |  |  |  |  |  |  |
| Observations | 3949 | 3949 | 3583 | 3583 | 1005 | 1005 |
| *** p<0.01, ** p<0.05, * p<0.1Standard errors in parentheses | | | |  |  |  |
