# Supplementary material for: Ageing under unequal circumstances: a cross-sectional analysis of the gender and socioeconomic patterning of functional limitations among the Southern European elderly
Source: Int J Equity Health. 2017 Oct 3;16:175. doi: 10.1186/s12939-017-0673-0 (PMC5627490; doi:10.1186/s12939-017-0673-0)
Supplement: Supplementary file 5 — Marginal effects for functional limitation from the multinomial model, by age group. Robustness check (II) of Table 3. Standard errors in parentheses *** p < 0.01, ** p < 0.05, * p < 0.1. Estimation of the same model as in Table 3, but setting a new cut-off for the dependent variable of functional limitation: moderate functionally limited if ADL + IADL is between one and three and severe functionally limited if ADL + IADL is equal or greater than four. (DOCX 14 kb) [file 12939_2017_673_MOESM5_ESM.docx]

|  | Mature adults (50-64) | | Elderly (65-79) | | Oldest old (80+) | |
| --- | --- | --- | --- | --- | --- | --- |
|  | (1) | (2) | (3) | (4) | (5) | (6) |
| VARIABLES | Moderately  limited | Severely  limited | Moderately  limited | Severely  limited | Moderately  limited | Severely  limited |
|  |  |  |  |  |  |  |
| Age | 0.002** | 0.000 | 0.012*** | 0.007*** | -0.007 | 0.033*** |
|  | (0.00) | (0.00) | (0.00) | (0.00) | (0.01) | (0.01) |
| Sex |  |  |  |  |  |  |
| Base category: Male |  |  |  |  |  |  |
| female | 0.036*** | -0.001 | 0.111*** | 0.012 | 0.157*** | 0.069 |
|  | (0.01) | (0.00) | (0.02) | (0.01) | (0.05) | (0.08) |
| Education level |  |  |  |  |  |  |
| Base category: No education | |  |  |  |  |  |
| Primary | -0.026 | -0.003 | -0.094*** | -0.051*** | -0.028 | -0.112 |
|  | (0.02) | (0.00) | (0.03) | (0.02) | (0.09) | (0.08) |
| Secondary | -0.060* | -0.012 | -0.085*** | -0.058*** | -0.121 | -0.158* |
|  | (0.03) | (0.01) | (0.03) | (0.02) | (0.09) | (0.08) |
| Tertiary | -0.034** | -0.012*** | -0.033 | -0.038 | 0.040 | -0.269*** |
|  | (0.02) | (0.00) | (0.03) | (0.02) | (0.16) | (0.07) |
| Subjective poverty |  |  |  |  |  |  |
| Base category: Not poor | |  |  |  |  |  |
| Poor | 0.039*** | 0.005 | 0.079*** | 0.062*** | 0.025 | 0.065 |
|  | (0.01) | (0.00) | (0.02) | (0.02) | (0.06) | (0.06) |
| Marital status |  |  |  |  |  |  |
| Based category: Not in a couple | |  |  |  |  |  |
| In a couple | 0.001 | -0.003 | 0.005 | -0.001 | -0.049 | -0.065 |
|  | (0.01) | (0.00) | (0.02) | (0.02) | (0.06) | (0.08) |
|  |  |  |  |  |  |  |
| Country dummies | |  |  |  |  |  |
| Spain | -0.027*** | 0.003 | -0.050** | -0.020 | 0.069 | -0.068 |
|  | (0.01) | (0.00) | (0.02) | (0.02) | (0.10) | (0.07) |
| Italy | -0.036*** | -0.009* | -0.013 | -0.024 | 0.118 | -0.057 |
|  | (0.01) | (0.01) | (0.03) | (0.02) | (0.10) | (0.09) |
|  |  |  |  |  |  |  |
| Observations | 3949 | 3949 | 3583 | 3583 | 1005 | 1005 |
| *** p<0.01, ** p<0.05, * p<0.1Standard errors in parentheses | | | |  |  |  |
