# Supplementary material for: Ageing under unequal circumstances: a cross-sectional analysis of the gender and socioeconomic patterning of functional limitations among the Southern European elderly
Source: Int J Equity Health. 2017 Oct 3;16:175. doi: 10.1186/s12939-017-0673-0 (PMC5627490; doi:10.1186/s12939-017-0673-0)
Supplement: Supplementary file 6 — Marginal effects from the logistic model of informal care, among those limited. Robustness check of Table 4. Standard errors in parentheses *** p < 0.01, ** p < 0.05, * p < 0.1. The new cut-offs for the level of limitation were: For columns (1) to (4) moderate functionally limited if ADL + IADL equals one and severe functionally limited if ADL + IADL is equal or greater than two; for columns (5) to (8) moderate functionally limited if ADL + IADL is between one and three and severe functionally limited if ADL + IADL is equal or greater than four. (DOCX 16 kb) [file 12939_2017_673_MOESM6_ESM.docx]

|  | (1) | (2) | (3) | (4) |  | (5) | (6) | (7) | (8) |
| --- | --- | --- | --- | --- | --- | --- | --- | --- | --- |
| VARIABLES | ES | IT | PT | All |  | ES | IT | PT | All |
|  |  |  |  |  |  |  |  |  |  |
| Level of limitation | |  |  |  |  |  |  |  |  |
| Base category: *Moderately limited* | | |  |  |  |  |  |  |  |
| Severely limited | 0.192*** | 0.306*** | 0.338*** | 0.270*** |  | 0.258*** | 0.356*** | 0.411*** | 0.329*** |
|  | (0.05) | (0.05) | (0.05) | (0.03) |  | (0.04) | (0.07) | (0.04) | (0.04) |
|  |  |  |  |  |  |  |  |  |  |
| Age | 0.011*** | 0.007** | 0.010*** | 0.009*** |  | 0.009*** | 0.007** | 0.005 | 0.008*** |
|  | (0.00) | (0.00) | (0.00) | (0.00) |  | (0.00) | (0.00) | (0.00) | (0.00) |
| Sex |  |  |  |  |  |  |  |  |  |
| Base category: *Male* | |  |  |  |  |  |  |  |  |
| Female | 0.073 | -0.145* | 0.076 | -0.049 |  | 0.074 | -0.076 | 0.003 | -0.017 |
|  | (0.06) | (0.08) | (0.08) | (0.05) |  | (0.06) | (0.08) | (0.07) | (0.05) |
| Education level | |  |  |  |  |  |  |  |  |
| Base category: *No education* | | |  |  |  |  |  |  |  |
| Primary | -0.003 | 0.210* | 0.151** | 0.080 |  | -0.004 | 0.245** | 0.094 | 0.086 |
|  | (0.06) | (0.11) | (0.08) | (0.07) |  | (0.05) | (0.10) | (0.07) | (0.06) |
| Secondary | -0.149** | 0.273** | 0.243** | 0.081 |  | -0.140* | 0.285*** | 0.216** | 0.075 |
|  | (0.07) | (0.13) | (0.11) | (0.07) |  | (0.07) | (0.11) | (0.09) | (0.06) |
| Tertiary | -0.088 | -0.018 | -0.000 | -0.064 |  | -0.077 | -0.051 | 0.119 | -0.067 |
|  | (0.11) | (0.17) | (0.18) | (0.11) |  | (0.12) | (0.20) | (0.15) | (0.13) |
| Subjective poverty | |  |  |  |  |  |  |  |  |
| Base category: *Not poor* | | |  |  |  |  |  |  |  |
| Poor | -0.004 | 0.027 | 0.026 | 0.008 |  | -0.024 | 0.033 | -0.006 | -0.004 |
|  | (0.05) | (0.07) | (0.07) | (0.04) |  | (0.05) | (0.07) | (0.06) | (0.04) |
| Employment status | |  |  |  |  |  |  |  |  |
| Base category: *Active* | |  |  |  |  |  |  |  |  |
| Inactive | 0.136 | 0.020 | 0.012 | 0.094 |  | 0.196* | -0.018 | 0.018 | 0.130 |
|  | (0.12) | (0.13) | (0.13) | (0.08) |  | (0.11) | (0.13) | (0.13) | (0.08) |
| Homemaker | 0.003 | 0.118 | 0.071 | 0.103 |  | 0.086 | 0.069 | 0.108 | 0.145 |
|  | (0.13) | (0.15) | (0.13) | (0.10) |  | (0.12) | (0.15) | (0.14) | (0.09) |
| Marital status | |  |  |  |  |  |  |  |  |
| Base category: *Not in a couple* | | |  |  |  |  |  |  |  |
| In a couple | 0.070 | 0.013 | -0.051 | 0.032 |  | 0.062 | -0.054 | -0.025 | -0.000 |
|  | (0.05) | (0.07) | (0.07) | (0.04) |  | (0.05) | (0.06) | (0.06) | (0.04) |
|  |  |  |  |  |  |  |  |  |  |
| Country dummies | |  |  |  |  |  |  |  |  |
| Spain |  |  |  | 0.050 |  |  |  |  | 0.059 |
|  |  |  |  | (0.05) |  |  |  |  | (0.04) |
| Italy |  |  |  | 0.107** |  |  |  |  | 0.129*** |
|  |  |  |  | (0.05) |  |  |  |  | (0.05) |
|  |  |  |  |  |  |  |  |  |  |
| Observations | 727 | 605 | 506 | 1838 |  | 727 | 605 | 506 | 1838 |
|  |  |  |  |  |  |  |  |  |  |
