# Supplementary material for: Ageing under unequal circumstances: a cross-sectional analysis of the gender and socioeconomic patterning of functional limitations among the Southern European elderly
Source: Int J Equity Health. 2017 Oct 3;16:175. doi: 10.1186/s12939-017-0673-0 (PMC5627490; doi:10.1186/s12939-017-0673-0)
Supplement: Supplementary file 7 — Coefficients of the negative binomial models of healthcare use. Robustness check (I) of Table 5. Standard errors in parentheses *** p < 0.01, ** p < 0.05, * p < 0.1. The new cut-offs for the level of limitation were: moderate functionally limited if ADL + IADL equals one and severe functionally limited if ADL + IADL is equal or greater than two. (DOCX 17 kb) [file 12939_2017_673_MOESM7_ESM.docx]

|  | In-patient days | | | | |  | Doctor consultations | | | |
| --- | --- | --- | --- | --- | --- | --- | --- | --- | --- | --- |
|  | (1) | | (2) | (3) | (4) |  | (5) | (6) | (7) | (8) |
| VARIABLES | PT | | IT | ES | All |  | PT | IT | ES | All |
|  |  | |  |  |  |  |  |  |  |  |
| Level of limitation |  | |  |  |  |  |  |  |  |  |
| Base category: *Non-limited* | | |  |  |  |  |  |  |  |  |
| Moderately Limited | -0.219 | | 1.159*** | 0.518 | 0.588** |  | 0.411*** | 0.348*** | 0.502*** | 0.382*** |
|  | (0.40) | | (0.39) | (0.47) | (0.27) |  | (0.10) | (0.13) | (0.15) | (0.08) |
| Severely Limited | 1.603*** | | 1.352*** | 2.003*** | 1.578*** |  | 0.655*** | 0.512*** | 0.483*** | 0.571*** |
|  | (0.26) | | (0.24) | (0.38) | (0.18) |  | (0.08) | (0.10) | (0.15) | (0.07) |
| Sex |  | |  |  |  |  |  |  |  |  |
| Base category: *Male* |  | |  |  |  |  |  |  |  |  |
| Female | -0.567** | | -0.093 | 0.308 | -0.238 |  | 0.187** | 0.097 | 0.362** | 0.153*** |
|  | (0.28) | | (0.28) | (0.30) | (0.20) |  | (0.08) | (0.07) | (0.15) | (0.05) |
| Age | -0.008 | | 0.039*** | 0.023 | 0.011 |  | -0.005 | 0.005 | 0.003 | 0.001 |
|  | (0.01) | | (0.01) | (0.02) | (0.01) |  | (0.00) | (0.00) | (0.01) | (0.00) |
| Education level |  | |  |  |  |  |  |  |  |  |
| Base category: *No* *education* | | |  |  |  |  |  |  |  |  |
| Primary | -0.041 | | 0.578 | 0.489 | -0.027 |  | 0.058 | 0.058 | 0.181 | 0.096 |
|  | (0.27) | | (0.46) | (0.54) | (0.23) |  | (0.08) | (0.12) | (0.18) | (0.07) |
| Secondary | -0.548* | | 0.402 | 1.146* | -0.283 |  | -0.092 | -0.130 | 0.199 | -0.069 |
|  | (0.31) | | (0.49) | (0.64) | (0.25) |  | (0.09) | (0.13) | (0.20) | (0.07) |
| Tertiary | -0.682 | | 0.512 | 0.890 | -0.199 |  | -0.038 | -0.209 | 0.495* | -0.025 |
|  | (0.54) | | (0.71) | (0.67) | (0.43) |  | (0.17) | (0.20) | (0.30) | (0.12) |
| Subjective poverty |  | |  |  |  |  |  |  |  |  |
| Base category: *Not poor* | | |  |  |  |  |  |  |  |  |
| Poor | 0.277 | | 0.126 | 0.653* | 0.287* |  | 0.102 | 0.171** | 0.011 | 0.133*** |
|  | (0.26) | | (0.21) | (0.33) | (0.17) |  | (0.07) | (0.07) | (0.17) | (0.05) |
| Employment status | |  |  |  |  |  |  |  |  |  |
| Base category: *Active* | | | |  |  |  |  |  |  |  |
| Inactive | 1.684*** | | 0.268 | 0.632* | 0.890*** |  | 0.535*** | 0.552*** | 0.219 | 0.516*** |
|  | (0.37) | | (0.35) | (0.38) | (0.25) |  | (0.11) | (0.10) | (0.18) | (0.07) |
| Homemaker | 1.811*** | | -0.009 | 0.483 | 0.729** |  | 0.327*** | 0.365*** | 0.354 | 0.338*** |
|  | (0.45) | | (0.45) | (0.57) | (0.30) |  | (0.11) | (0.12) | (0.30) | (0.08) |
| Marital status |  | |  |  |  |  |  |  |  |  |
| Base category: *Not in couple* | | |  |  |  |  |  |  |  |  |
| In a couple | -0.372 | | 0.229 | -0.099 | -0.018 |  | 0.145** | 0.045 | 0.046 | 0.081 |
|  | (0.26) | | (0.23) | (0.37) | (0.18) |  | (0.07) | (0.08) | (0.12) | (0.05) |
|  |  | |  |  |  |  |  |  |  |  |
| Country dummies |  | |  |  |  |  |  |  |  |  |
| Spain FE |  | |  |  | 0.182 |  |  |  |  | 0.400*** |
|  |  | |  |  | (0.23) |  |  |  |  | (0.09) |
| Italy FE |  | |  |  | 0.420* |  |  |  |  | 0.559*** |
|  |  | |  |  | (0.23) |  |  |  |  | (0.09) |
| Observations | 3213 | | 3385 | 1906 | 8504 |  | 3204 | 3373 | 1884 | 8,461 |
|  |  | |  |  |  |  |  |  |  |  |
| Observations *** p<0.01, ** p<0.05, * p<0.1 |  | |  |  |  |  |  |  |  |  |
