# Supplementary material for: Ageing under unequal circumstances: a cross-sectional analysis of the gender and socioeconomic patterning of functional limitations among the Southern European elderly
Source: Int J Equity Health. 2017 Oct 3;16:175. doi: 10.1186/s12939-017-0673-0 (PMC5627490; doi:10.1186/s12939-017-0673-0)
Supplement: Supplementary file 8 — Coefficients of the negative binomial models of healthcare use. Robustness check (II) of Table 5. Standard errors in parentheses *** p < 0.01, ** p < 0.05, * p < 0.1. The new cut-offs for the level of limitation were: moderate functionally limited if ADL + IADL is between one and three and severe functionally limited if ADL + IADL is equal or greater than four. (DOCX 18 kb) [file 12939_2017_673_MOESM8_ESM.docx]

|  | In-patient days | | | | |  | Doctor consultations | | | |
| --- | --- | --- | --- | --- | --- | --- | --- | --- | --- | --- |
|  | (1) | | (2) | (3) | (4) |  | (5) | (6) | (7) | (8) |
| VARIABLES | PT | | IT | ES | All |  | PT | IT | ES | All |
|  |  | |  |  |  |  |  |  |  |  |
| Level of limitation |  | |  |  |  |  |  |  |  |  |
| Base category: *Non-limited* | | |  |  |  |  |  |  |  |  |
| Moderately Limited | 0.898*** | | 1.189*** | 0.897*** | 0.990*** |  | 0.499*** | 0.458*** | 0.530*** | 0.485*** |
|  | (0.28) | | (0.27) | (0.34) | (0.19) |  | (0.07) | (0.10) | (0.13) | (0.06) |
| Severely Limited | 1.710*** | | 1.459*** | 2.598*** | 1.733*** |  | 0.697*** | 0.435*** | 0.392** | 0.540*** |
|  | (0.31) | | (0.29) | (0.57) | (0.21) |  | (0.10) | (0.13) | (0.18) | (0.08) |
| Sex |  | |  |  |  |  |  |  |  |  |
| Base category: *Male* |  | |  |  |  |  |  |  |  |  |
| Female | -0.544* | | -0.075 | 0.248 | -0.216 |  | 0.186** | 0.098 | 0.371** | 0.155*** |
|  | (0.28) | | (0.29) | (0.30) | (0.20) |  | (0.08) | (0.07) | (0.15) | (0.05) |
| Age | -0.011 | | 0.039*** | 0.019 | 0.012 |  | -0.006 | 0.006 | 0.004 | 0.002 |
|  | (0.01) | | (0.01) | (0.02) | (0.01) |  | (0.00) | (0.00) | (0.01) | (0.00) |
| Education level |  | |  |  |  |  |  |  |  |  |
| Base category: *No* *education* | | |  |  |  |  |  |  |  |  |
| Primary | -0.051 | | 0.620 | 0.301 | 0.023 |  | 0.059 | 0.066 | 0.169 | 0.092 |
|  | (0.28) | | (0.44) | (0.54) | (0.24) |  | (0.08) | (0.12) | (0.18) | (0.07) |
| Secondary | -0.580* | | 0.438 | 0.893 | -0.250 |  | -0.090 | -0.122 | 0.186 | -0.074 |
|  | (0.32) | | (0.47) | (0.63) | (0.26) |  | (0.09) | (0.13) | (0.20) | (0.07) |
| Tertiary | -0.698 | | 0.553 | 0.782 | -0.144 |  | -0.038 | -0.202 | 0.482 | -0.031 |
|  | (0.55) | | (0.69) | (0.67) | (0.43) |  | (0.17) | (0.20) | (0.30) | (0.12) |
| Subjective poverty |  | |  |  |  |  |  |  |  |  |
| Base category: *Not poor* | | |  |  |  |  |  |  |  |  |
| Poor | 0.286 | | 0.126 | 0.418 | 0.298* |  | 0.102 | 0.175** | 0.015 | 0.135*** |
|  | (0.26) | | (0.21) | (0.31) | (0.17) |  | (0.07) | (0.07) | (0.17) | (0.05) |
| Employment status | |  |  |  |  |  |  |  |  |  |
| Base category: *Active* | | | |  |  |  |  |  |  |  |
| Inactive | 1.728*** | | 0.268 | 0.657* | 0.876*** |  | 0.539*** | 0.541*** | 0.217 | 0.511*** |
|  | (0.37) | | (0.35) | (0.39) | (0.25) |  | (0.11) | (0.10) | (0.18) | (0.07) |
| Homemaker | 1.809*** | | -0.018 | 0.310 | 0.685** |  | 0.339*** | 0.354*** | 0.349 | 0.332*** |
|  | (0.46) | | (0.45) | (0.55) | (0.30) |  | (0.11) | (0.12) | (0.30) | (0.08) |
| Marital status |  | |  |  |  |  |  |  |  |  |
| Base category: *Not in couple* | | |  |  |  |  |  |  |  |  |
| In a couple | -0.383 | | 0.224 | -0.051 | -0.039 |  | 0.143** | 0.038 | 0.040 | 0.074 |
|  | (0.27) | | (0.23) | (0.36) | (0.18) |  | (0.07) | (0.08) | (0.12) | (0.05) |
|  |  | |  |  |  |  |  |  |  |  |
| Country dummies |  | |  |  |  |  |  |  |  |  |
| Spain FE |  | |  |  | 0.261 |  |  |  |  | 0.403*** |
|  |  | |  |  | (0.22) |  |  |  |  | (0.09) |
| Italy FE |  | |  |  | 0.466** |  |  |  |  | 0.563*** |
|  |  | |  |  | (0.22) |  |  |  |  | (0.09) |
| Observations | 3213 | | 3385 | 1906 | 8504 |  | 3204 | 3373 | 1884 | 8461 |
|  |  | |  |  |  |  |  |  |  |  |
